# Side effects related to systemic cancer treatment: are we changing the Promethean experience with molecularly targeted therapies?

**DOI:** 10.3747/co.v15i4.362

**Published:** 2008-08

**Authors:** Carlo De Angelis

**Affiliations:** Clinical Pharmacy Coordinator–Oncology, Odette Cancer Centre, Sunnybrook Health Sciences Centre, Toronto, Ontario

For having stolen fire and given it to mankind, Prometheus was punished by Zeus by being chained to the side of Mount Caucasus, where, every day, an eagle would eat away at his liver. Prometheus’s liver would regenerate itself overnight, ready to be eaten again with the coming dawn. Despite significant advances in supportive care, modern day chemotherapy can still be a Promethean experience for many cancer patients.

The use of cytotoxic chemotherapy to treat cancer dates back to the 1940s, and although progress in the ability to treat various types of cancer has been significant, much of that progress has relied on the introduction of new cytotoxic agents with novel, but non-selective mechanisms of action 1. The availability of supportive care agents such as 5-hydroxytryptamine_3_ (5-HT_3_) receptor antagonists, neurokinin 1 (NK_1_) receptor in- hibitors, and growth factors such as filgrastim and eryth-ropoietin have also allowed treatment advances to be made by providing tools to better manage the side effects of chemotherapy, facilitating our ability to optimize the delivery of traditional cytotoxic agents, to push the boundaries of their steep dose–response curve, and to widen the narrow therapeutic index.

However, with the exception of anti-hormonal therapy for breast and prostate cancer, the ability to truly exploit the differences between cancer cells and normal cells was realized only in 2001, with the introduction of imatinib, the first molecularly targeted agent for the treatment of chronic myeloid leukemia positive for the Philadelphia chromosome 1. What makes today’s molecularly targeted therapies different from the more traditional cytotoxic agents is that they have been developed with a predefined extracellular or intracellular target or pathway in mind. These pathways have been identified as functioning in an aberrant manner in cancer cells relative to normal cells. To date, agents have been developed that disrupt pathways controlling cancer cell growth, differentiation, transcription, or angiogenesis. These agents also tend to have a reversible pharmacologic effect, to be cytostatic rather than cytotoxic, and to be most often given on a regular ongoing daily schedule rather than in cycles 2,3.

The currently available molecularly targeted therapies fall into two broad categories: monoclonal antibodies that target cell surface proteins, and small-molecule kinase inhibitors that inhibit intracellular signalling pathways. From the perspective of mechanism of action, the first generation of agents either interact with epidermal growth factor pathways or inhibit angiogenesis. The newer multi-targeted agents (some currently available, and many more in development) affect multiple intracellular kinase targets 4–7. A discussion comparing modes of action and clinical efficacies of currently available molecularly targeted therapies (rituximab, trastuzumab, bevacizumab, imatinib, erlotinib, sunitinib, and sorafenib to name a few) is beyond the scope of this editorial, but recent reviews are readily available 4,8.

In the rush to bring molecularly targeted therapies into day-to-day clinical practice, the side effects associated with these agents—used either alone or in combination with traditional cytotoxic agents—have received little attention. It had been postulated that, because of their increased selectivity for cancer cells, these agents would be less toxic than the traditional cytotoxic agents. However, it has been learned that these agents can indeed cause toxicities in patients—perhaps not surprisingly, in retrospect, because they target key signalling pathways for cellular growth and development. These toxicities are, for the most part, different from the toxicities of traditional cytotoxic agents, but they can nevertheless lead to dose reductions and delays and reduced quality of life for oncology patients.

In addition to familiar side effects such as diarrhea, mucosal membrane toxicity, palmer–plantar erythrodysesthesia, and infusion reactions (for the monoclonal antibodies), the targeted agents cause relatively unique side effects, including proteinuria, hypertension, and skin reactions (acneiform rash, dry skin, nail changes, hair depigmentation) 4,9,10. Relative to the body of literature supporting the clinical efficacy of molecularly targeted therapies, information regarding their side effects is lacking. These unique side effects are no less distressing to patients, and they affect quality of life as much as the side affects associated with traditional cytotoxic therapy. Indeed, when targeted therapies are used in combination with traditional cytotoxic treatment, practitioners are adding to the range of toxicities experienced by patients.

The advent of new molecularly targeted therapies brought with it the belief that the oncology community, like Heracles (“Hercules” in Roman mythology), who freed Prometheus, would free patients from the cyclical experience of the side effects associated with traditional cytotoxic chemotherapeutic agents. To a certain extent, this expectation has been realized, but these agents pose other challenges that require vigilance with respect to treatment-related side effects.

The name Prometheus means “forethought”; as advocates for patients, we must act with foresight and learn to anticipate treatment-related side effects, implementing strategies to prevent their occurrence and acting to mitigate the severity of these side effects when they do occur.

For the molecularly targeted therapies, the challenges that lie ahead include characterization of their toxicity profile (onset, severity, duration) in the broader cancer patient population, development of instruments that can be used in day-to-day practice by patients or by health care practitioners to assess the occurrence of side effects, systematic evaluation of strategies to prevent or manage treatment-related side effects, and development of tools that can help to identify patients at risk for development of side effects. Only then can the true potential of individualized anticancer therapy with molecularly targeted therapies be realized—and the chains of Prometheus broken once and for all for the sake of our patients.
